# Genomic characteristics of pancreatic squamous cell carcinoma, an investigation by using high throughput sequencing after in-solution hybrid capture

**DOI:** 10.18632/oncotarget.14678

**Published:** 2017-01-16

**Authors:** Meng-Dan Xu, Shu-Ling Liu, Yi-Zhong Feng, Qiang Liu, Meng Shen, Qiaoming Zhi, Zeyi Liu, Dong-Mei Gu, Jie Yu, Liu-Mei Shou, Fei-Ran Gong, Qi Zhu, Weiming Duan, Kai Chen, Junning Zhang, Meng-Yao Wu, Min Tao, Wei Li

**Affiliations:** ^1^ Department of Oncology, The First Affiliated Hospital of Soochow University, Suzhou 215006, China; ^2^ Department of Radiation Oncology, The First Affiliated Hospital of Soochow University, Suzhou 215006, China; ^3^ Department of Pathology, The Second Affiliated Hospital of Soochow University, Suzhou 215006, China; ^4^ Department of Pathology, Renji Hospital Affiliated to Shanghai Jiao Tong University School of Medicine, Shanghai 200000, China; ^5^ Department of General Surgery, The First Affiliated Hospital of Soochow University, Suzhou 215006, China; ^6^ Department of Respiratory Medicine, The First Affiliated Hospital of Soochow University, Suzhou 215006, China; ^7^ Department of Pathology, The First Affiliated Hospital of Soochow University, Suzhou 215006, China; ^8^ Department of Oncology, The First Affiliated Hospital of Zhejiang Chinese Medicine University, Hangzhou 310006, China; ^9^ Department of Hematology, The First Affiliated Hospital of Soochow University, Suzhou 215006, China; ^10^ Xi'an Tianlong Science and Technology Co., Ltd., Xi'an 710018, China; ^11^ PREMED Key Laboratory for Precision Medicine, Soochow University, Suzhou 215021, China; ^12^ Jiangsu Institute of Clinical Immunology, Suzhou 215006, China; ^13^ Institute of Medical Biotechnology, Soochow University, Suzhou 215021, China

**Keywords:** pancreatic squamous cell carcinoma, pancreatic adenocarcinoma, high throughput sequencing (HTS), in-solution hybrid capture

## Abstract

Squamous cell carcinoma (SCC) of pancreas is a rare histotype of pancreatic ductal carcinoma which is distinct from pancreatic adenocarcinoma (AC). Although there are standard treatments for pancreatic AC, no precise therapies exist for pancreatic SCC. Here, we screened 1033 cases of pancreatic cancer and identified 2 cases of pure SCC, which were pathologically diagnosed on the basis of finding definite intercellular bridges and/or focal keratin peal formation in the tumor cells. Immunohistochemistry assay confirmed the positive expression of CK5/6 and p63 in pancreatic SCC. To verify the genomic characteristics of pancreatic SCC, we employed in-solution hybrid capture targeting 137 cancer-related genes accompanied by high throughput sequencing (HTS) to compare the different genetic variants in SCC and AC of pancreas. We compared the genetic alterations of known biomarkers of pancreatic adenocarcinoma in different pancreatic cancer tissues, and identified nine mutated genes in SCC of pancreas: C7orf70, DNHD1, KPRP, MDM4, MUC6, OR51Q1, PTPRD, TCF4, TET2, and nine genes (ABCB1, CSF1R, CYP2C18, FBXW7, ITPA, KIAA0748, SOD2, SULT1A2, ZNF142) that are mutated in pancreatic AC. This study may have taken one step forward on the discovery of potential biomarkers for the targeted treatment of SCC of the pancreas.

## INTRODUCTION

Pancreatic cancer is an aggressive malignant tumor of digestive tract and a leading cause of deaths related to cancer worldwide [1, 2]. 85%–90% of pancreatic cancers are pancreatic ductal carcinoma, and most of which are adenocarcinoma (AC). Squamous cells do not exist in the normal pancreatic tissues, hence the pathogenesis of squamous cell carcinoma (SCC) remains unclear. Although SCC of the pancreas is rare, accounting for less than 1% of all cases of pancreatic neoplasms [3], incidence rates for this subtype are increasing in the past decade [4].

Though SCC arising in other solid tumors are proved to be chemosensitive or radiosensitive [5], the prognosis of SCC in pancreas is worse than other subtype of pancreatic cancer according to previous studies [4, 6]. In addition, although there are already standard treatments for pancreatic AC, the low incidence makes pancreatic SCC a mystery, and there are still no personal therapeutics against this rare pancreatic cancer type [3]. Therefore, exploring the characteristics of pancreatic SCC is necessary for developing targeted strategies.

Recently, a population-based study reported the epidemiology of pancreatic SCCs [4]. Moreover, another investigation revealed the gene expression patterns of pancreatic SCCs [6]. These new progressions provided important values for understanding this rare neoplasm. The rapid development of next generation sequencing (NGS) technologies has made a revolution to cancer research [7–10]. NGS technology has made it possible to detect the genetic variants including single-nucleotide variant (SNV) and Insertion and Deletion (InDel) throughout the human genome with the high throughput sequencing (HTS) methodologies. In addition, the formalin fixed paraffin embedded (FFPE) samples from pathological archives opened up the gate of the treasure that possessed abundant patient samples for NGS sequencing. In the present article, we perfomed Illumina paired-end sequencing after in-solution hybrid capture targeting 137 genes (Supplementary Table 1) related to somatic alterations in cancer [11–13], to identify the genomic characterristics of pancreatic SCC. These 137 genes include tumor suppressors, oncogenes, and prognostic markers that frequently possess mutation in cancers. The differential mutated genes have been discovered in pancreatic AC and SCC, respectively, which could give evidences for future researches on pathogenesis and treatment of pancreatic SCC.

## RESULTS

### Epidemiology of pancreatic SCC in Chinese population

Recently, Makarova-Rusher OV et. al. performed the first population-based study to report on the epidemiology of primary pancreatic SCC by screening 214 patients with SCC from 2000 to 2012 in the United States [4]. Based on their study, SCCs accounted for only 0.2 % of pancreatic cancers in the United States. To find out this proportion in Chinese population, we screened 1033 pancreatic cancer cases in the First Affiliated Hospital of Soochow University (Suzhou, China), the Second Affiliated Hospital of Soochow University (Suzhou, China), and Renji Hospital (Shanghai, China). During December, 1998 to July, 2016, there were 405 cases diagnosed as pancreatic cancer in the First Affiliated Hospital of Soochow University, while only one of them were diagnosed as SCC and six cases were diagnosed as ASC. From August, 2008 to August, 2016, 169 patients were diagnosed as pancreatic cancer in the Second Affiliated Hospital of Soochow University, but none of them was SCC. 459 patients with pancreatic cancer were admitted to Renji Hospital during August, 2008 to August, 2016. One of them was diagnosed as pure SCC, and 16 were confirmed to be ASC. Therefore, in all these 1033 cases, only 2 were diagnosed as pure SCC, making the proportion of SCC in the screened Chinese population was only 0.19%.

### Pathological confirmation of pancreatic SCC

Since there are still no comparison between genomic characterristics of pancreatic SCC and AC, we carried out high throughput sequencing (HTS) after in-solution hybrid capture by using 2 SCC, 2 ASC and 4 AC FFPE tissue samples. Before HTS, histological reconfirmation was firstly performed.

**Figure 1:**
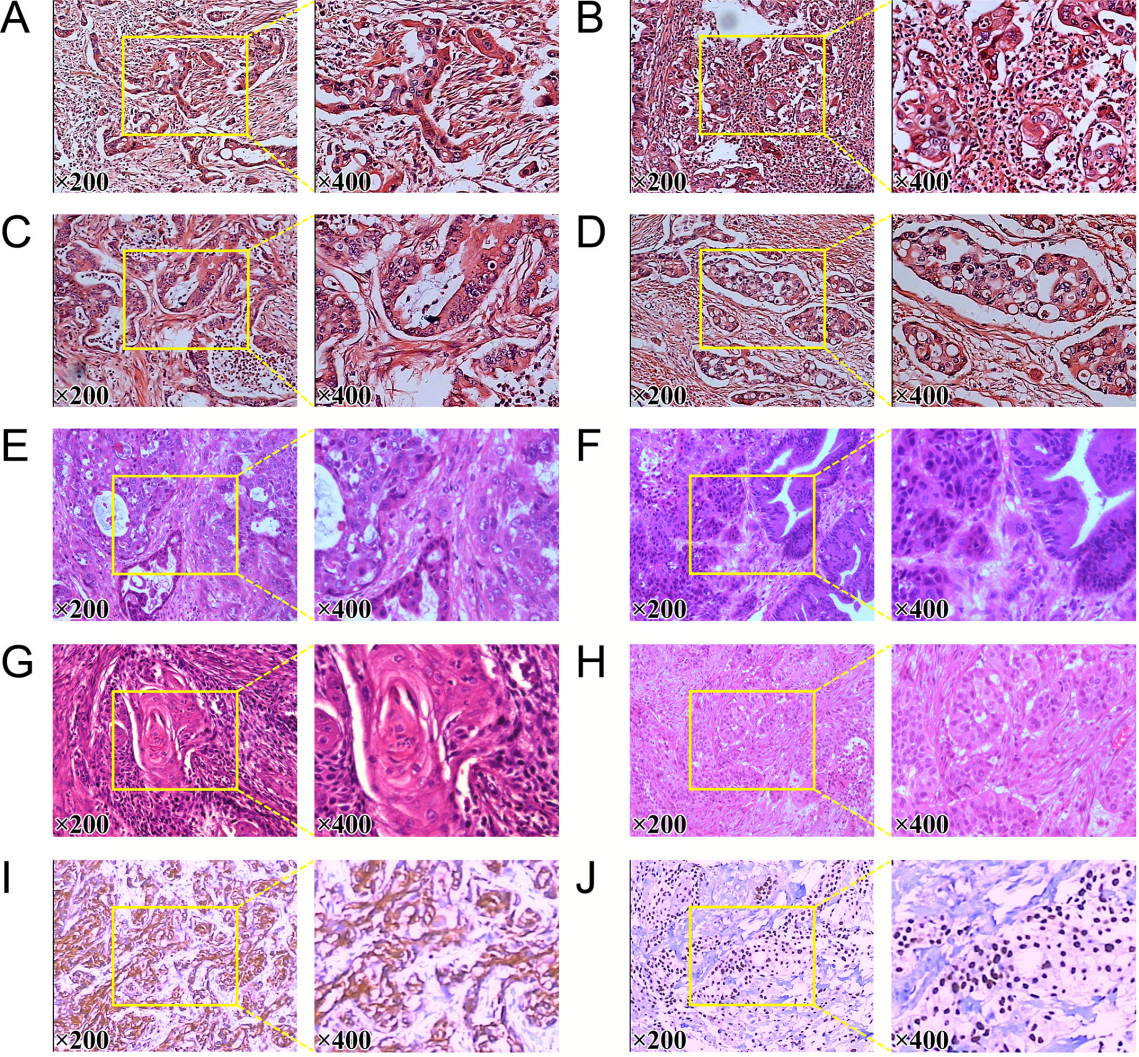
Pathological reconfirmation of pancreatic SCC (**A**) to (**D**) histological reconfirmation of four pancreatic AC samples. (**E**) and (**F**) Histology of two pancreatic ASC samples. (**G**) and (**H**) histological reconfirmation of two pancreatic SCC samples. (**I**) Expression of CK5/6 in pancreatic SCC sample. (**J**) Expression of P63 in pancreatic SCC sample.

As shown in Figure 1A–1H, pathological examination of pancreatic SCCs and ASCs revealed various grades of differentiated cancer cells. As compared with AC, patients with SCC had poorly differentiated histology, in consisting with previous studies [4]. Microscopically, SCC of pancreas was characterized by irregulard infiltrative nests or sheets of polygonal cells with distinct cellular borders, intercellular bridges, eosinophilic cytoplasm, and/or focal keratin peal formation in the tumors. Microscopic examination also documented that there was no evidence of glandular differentiation in pure SCC. Besides, the tumors of SCC shared a common characteristic of chronic inflammatory change around the tumor. Histological examination identified marked infiltration of chronic inflammatory cells and fibrosis in the background of the tumor. Besides, in higher power image, we can see a prominent desmoplastic response in stroma clearly. Calcifications scattered along the duct of Wirsung was observed as well. Previous immunohistochemical study showed that the pancreatic SCC cells were positive for CK5/6, p40, p63, and CK19, which were the superior markers for SCC [5, 14, 15]. As shown in Figure 1I and 1J, CK5/6 and p63 were positively expressed in pancreatic SCC samples, in consisting with previous studies [5, 14, 15].

### Sequence capture of FFPE pancreatic cancer tissues

The sequenced fastq output resulted in approximately 3.5 million reads for each of the FFPE pancreatic cancer tissues. Out of the raw reads, around 3.05 million reads for the SCCs, 2.99 millions reads for ASCs and approximately 3.03 million reads for the ACs were uniquely mapped to the human genome assembly hg19. The number of reads uniquely mapped to the target region was ~0.13 million for SCCs, ~0.16 million for ASCs and about 0.29 million for ACs. The percentage of the target region coverage was –53% for each sample of pancreatic cancer tissues (Table 1).

**Table 1:** Comparison of sequencing read data for FFPE pancreatic SCC, ASC and AC tissues

Sample	Squamous Cell Carcinoma (SCC)	Adenosquamous Carcinoma (ASC)	Adenocarcinoma (AC)
Initial bases on target	1091949	1091949	1091949
Total effective reads	3507289 ± 179140	3413500 ± 111295	3396153 ± 463643
Total effective yield(Mb)	336.78 ± 50.37	355.95 ± 2.19	389.07 ± 25.53
Number of reads uniquely mapped to target	130133 ± 6578	167451 ± 42792	289486 ± 56272
Number of reads uniquely mapped to genome	3050910 ± 149595	2995950 ± 89141	3026670 ± 415926
Fraction of effective bases on target	2.80% ± 0.14%	3.55% ± 0.77%	6.18% ± 1.01%
Fraction of uniquely mapped on target	4.30% ± 0.00%	5.60%±1.27%	9.60% ± 1.49%
Average sequencing depth on target	9.66 ± 0.50	11.97 ± 2.87	20.45 ± 3.58
Mismatch rate in target region	0.51% ± 0.04%	0.50% ± 0.01%	0.45% ± 0.03%
Mismatch rate in all effective sequence	0.81% ± 0.14%	0.87% ± 0.04%	0.73% ± 0.07%
Base covered on target	583643 ± 16402	579505 ± 9532	575904 ± 9634
Coverage of target region	53.45% ± 1.48%	53.10% ± 0.85%	52.75% ± 0.87%
Fraction of target covered with at least 20x	21.40% ± 1.97%	28.40% ± 7.64%	36.90% ± 1.45%
Fraction of target covered with at least 10x	38.65% ± 0.21%	39.20% ± 1.83%	41.10% ± 0.64%
Fraction of target covered with at least 4x	44.60% ± 0.98%	43.75% ± 0.63%	44.47% ± 0.81%
Mapping rate	87.77% ± 14.93%	98.54% ± 0.30%	99.26% ± 0.20%
Duplicate rate	73.71% ± 0.28%	72.22% ± 0.78%	69.93% ± 1.31%

### SNV (single-nucleotide variant) analysis

**Figure 2:**
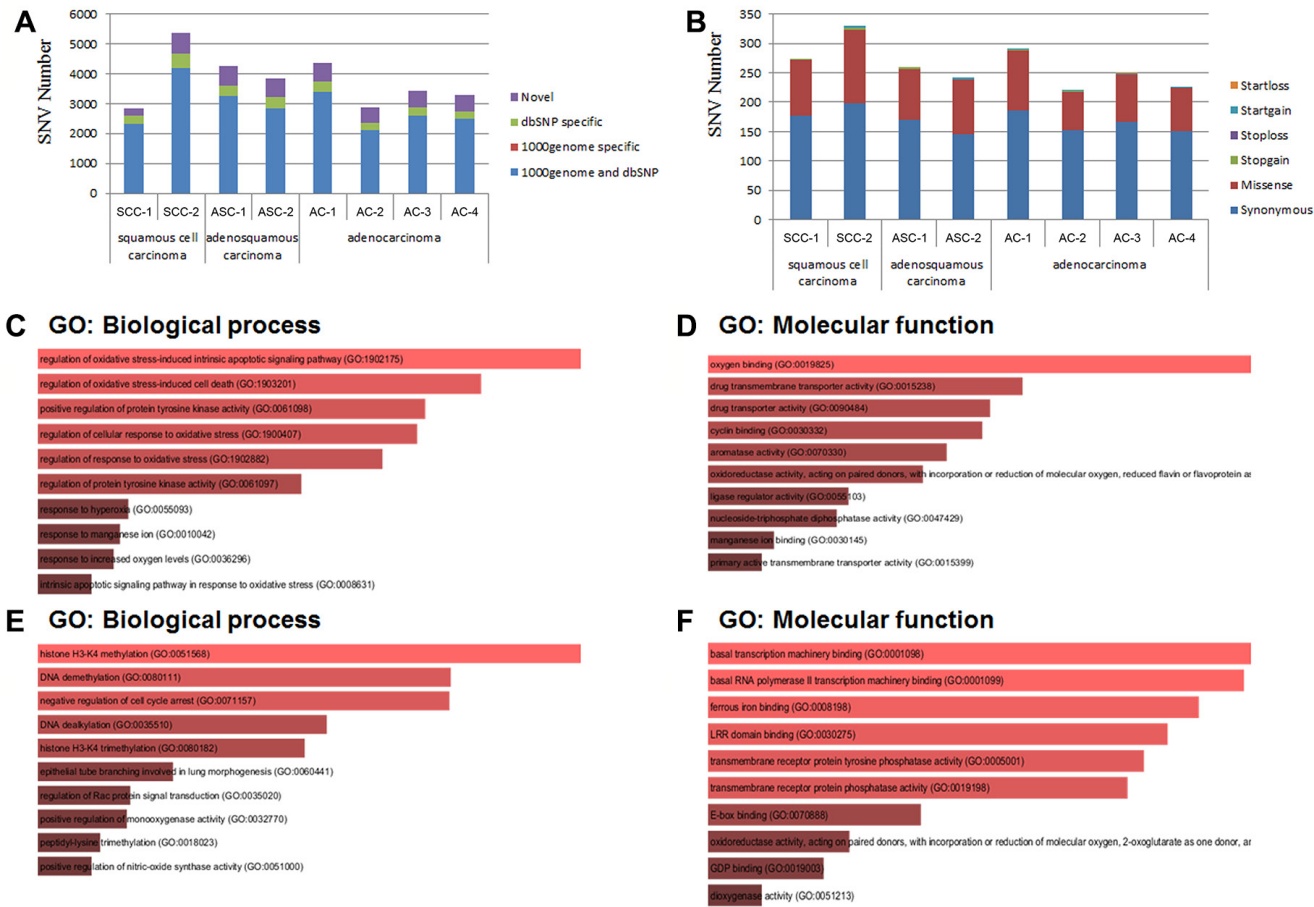
SNV (single-nucleotide variant) analysis (**A**) Distribution of total SNVs for each sample in 1000 genome and dbSNP. 1000 genome and dbSNP: the number of SNVs that fall into both 1000 genome and dbSNP; 1000 genome specific: the number of SNVs that only fall into 1000 genome, not in dbSNP; dbSNP specific: the number of SNVs that only fall into dbSNP, not in 1000 genome; The SNVs that fall into neither 1000 genome nor dbSNP were considered as “novel”. (**B**) Distribution of total SNVs that fall into the different categories leading to different genetic characters. Startloss: a nonsynonymous SNV that leads to the immediate elimination of start codon at the variant site; Startgain: a nonsynonymous SNV that leads to the immediate creation of start codon at the variant site; Stoploss: a nonsynonymous SNV that leads to the immediate elimination of stop codon at the variant site; Stopgain: Stoploss: a nonsynonymous SNV that leads to the immediate creation of stop codon at the variant site; Missense: a single nucleotide change that cause an amino acide change; Synonymous: a single nucleotide change that does not cause an amino acid change. (**C**) and (**D**) Gene Ontology (GO) analysis of SNVs specific in adenocarcinoma of pancreatic tissues. (C) Gene Ontology (GO): Biological process analysis; (D) Gene Ontology (GO): Molecular function analysis. (**E**) and (**F**) Gene Ontology (GO) analysis of SNVs specific in squamous cell carcinoma of pancreatic tissues. (E) Gene Ontology (GO): Biological process analysis; (F) Gene Ontology (GO): Molecular function analysis.

We have discovered a total of 2840 and 5369 SNVs for the two samples of SCC respectively, 4275 and 3837 SNVs for the two samples of ASC respectively, and 4354, 2888, 3442 and 3276 SNVs for the four samples of AC respectively. Approximately 9% to 18.49% detected SNVs fall into neither of the two categories: dbSNP and 1000 genome, which are the databases of SNPs (single-nucleotide polymorphisms) from normal healthy human beings (Figure 2A, Table 2). Therefore, 257~686 novel SNVs have been found in each sample of the pancreatic cancer tissues, which accounts for 9% to 18.4% of the total called SNVs from each sample (Table 2). In addition, most of the SNVs for each sample were homozygous genotypes, only a percentage ranging from 23.11% to 32.76% was heterozygous (Table 2). Among the total SNVs for each sample, about 6.37% to 10.53% of SNVs were detected in the coding region of genes (Table 2). We also detected similar rate of transition to transversion among SCCs, ASCs and ACs (Table 2). Importantly, around 69~131 non-synonymous SNVs have been discovered in each FFPE sample (Figure 2B, Table 2).

**Table 2:** Summary of single nucleotide variants (SNVs) for FFPE pancreatic SCC, ASC and AC tissues

Sample	Squamous Cell Carcinoma (SCC)		Adenosquamous Carcinoma (ASC)		Adenocarcinoma (AC)			
	SCC-1	SCC-2	ASC-1	ASC-2	AC-1	AC-2	AC-3	AC-4
Total	2840	5369	4275	3837	4354	2888	3442	3276
1000genome and dbsnp	2323	4194	3257	2833	3397	2101	2590	2492
1000genome specific	9	7	6	9	9	7	8	10
dbSNP specific	251	482	342	371	324	246	279	240
dbSNP rate	90.63%	87.09%	84.19%	83.50%	85.46%	81.27%	83.35%	83.39%
Novel	257 (9%)	686 (12.78%)	670 (15.67%)	624 (16.26%)	624 (14.33%)	534 (18.49%)	565 (16.41%)	534 (16.30%)
Hom	2057	4128	3254	2841	3194	1942	2539	2242
Het	783 (27.57%)	1241 (23.11%)	1021 (23.88%)	996 (25.95%)	1160 (26.64%)	946 (32.76%)	903 (26.23%)	1034 (31.56%)
Synonymous	177	198	170	146	186	153	166	150
Missense	96	126	87	93	102	65	82	74
Stopgain	1	2	3	0	1	1	0	0
Stoploss	0	0	0	0	0	0	0	0
Startgain	0	5	0	3	3	3	1	2
Startloss	0	0	0	0	0	0	1	0
SIFT	20	20	18	20	22	15	13	16
Ti/Tv	2.2719	2.2441	2.4728	2.2683	2.4042	2.2633	2.332	2.3738
dbSNP Ti/Tv	2.3691	2.3069	2.4908	2.2694	2.4169	2.2643	2.3477	2.4322
Novel Ti/Tv	1.5446	1.8824	2.401	2.267	2.3191	2.2561	2.2659	2.1047

In order to compare the genotype of SCC, ASC and AC, we searched for the different SNVs for the well-known pancreatic AC biomarkers (Table 3). All these pancreatic cancer tissues possessed the same TP53 mutation site (Table 3). Both SCC and AC samples contained KRAS mutation site (Table 3). We have also found mutation sites of several genes exclusively existing in SCCs, including SMAD4 and IGF1R, while some other mutation sites occurred only in pancreatic ACs, such as TERT, BRCA2, FGFR1, and FGFR4 (Table 3).

**Table 3:** SNVs of major pancreatic ductal adenocarcinoma markers for SCC, ASC and AC

Gene	Adenocarcinoma (AC)	adenosquamous carcinoma (ASC)	squamous cell carcinoma (SCC)
KRAS	NM_033360:p.Gly12Arg	–	NM_033360:p.Gly12Asp
TP53	NM_000546:p.Pro72Arg	NM_000546:p.Pro72Arg	NM_000546:p.Pro72Arg
SMAD4	–	NM_005359:p.Pro292Ser	NM_005359:p.Arg496His
TERT	NM_198253:p.Gln384Arg	–	–
IGF1R	–	NM_000875:p.Gly7Glu	NM_000875:p.Pro842Ser
BRCA2	NM_000059:p.Val2466Ala NM_000059:p.Asn372HisNM_000059:p.Asn289HisNM_000059:p.Asn991AspNM_000059:p.Thr2471SerNM_000059:p.Ile3363MetNM_000059:p.Ser3366AsnNM_000059:p.Val2466Ala	NM_000059:p.Cys315Ser NM_000059:p.Val2466AlaNM_000059:p.Asn289HisNM_000059:p.Asn991AspNM_000059:p.Asp2438Asn	NM_000059:p.Asn372His NM_000059:p.Val2466Ala
EGFR	NM_005228:p.Arg521Lys NM_005228:p.Leu861Gln	NM_005228:p.Arg521Lys NM_005228:p.Leu861Gln	NM_005228:p.Arg521Lys
FGFR1	NM_001174067:p.Ala392Val	–	–
FGFR4	NM_002011:p.Val10Ile NM_002011:p.Pro136LeuNM_002011:p.Gly388ArgNM_002011:p.Thr179Ala	NM_002011:p.Pro136Leu NM_002011:p.Gly388Arg	NM_002011:p.Pro136Leu NM_002011:p.Gly388Arg

We then selected those mutated genes that did not occur in pure SCCs. A total of nine mutated genes were found in ASCs and ACs, including ABCB1, CSF1R, CYP2C18, FBXW7, ITPA, KIAA0748, SOD2, SULT1A2, ZNF142. Among these nine mutated genes, four genes (ABCB1, CYP2C18, SOD2, and ZNF142) were discovered to possess SNVs only in ACs (Table 4). Gene Ontology (GO) analysis showed that these nine specific mutated genes were involved in the following main biological processes: regulation of oxidative stress-induced intrinsic apoptotic signaling pathway (GO:1902175) and cell death (GO:1903201) (Figure 2C) and in the following main molecular functions: oxygen binding (GO:0019825), drug transmembrane transporter activity (GO: 0015238) (Figure 2D).

**Table 4:** Summary of specific mutated genes in pancreatic AC tissues

Gene	Squamous Cell Carcinoma (SCC)	Adenosquamous Carcinoma (ASC)	Adenocarcinoma (AC)
ABCB1	0	0	2
CSF1R	0	1	5
CYP2C18	0	0	3
FBXW7	0	2	1
ITPA	0	0	2
KIAA0748	0	1	3
SOD2	0	0	2
SULT1A2	0	1	2
ZNF142	0	0	2

In addition, we also found nine genes (C7orf70, DNHD1, KPRP, MDM4, MUC6, OR51Q1, PTPRD, TCF4, and TET2) that possess no mutation in ACs. Among these nine mutated genes, four genes (DNHD1, OR51Q1, PTPRD, and TCF4) were discovered to possess SNVs exclusively in SCCs (Table 5). The analysis of Gene Ontology (GO) discovered that these nine mutated genes were involved in the following main biological processes: histone H3K4 methylation (GO:0051568), DNA methylation (GO: 0080111) and negative regulation of cell cycle arrest (GO: 0071157) (Figure 2E). For the GO category of molecular functions, these nine mutated genes were mainly involved in basal transcription machinery binding (GO: 0001098), ferrous iron binding (GO:0008198) and LRR domain binding (GO: 0030275) (Figure 2F).

**Table 5:** Summary of specific mutated genes in pancreatic SCC and ASC tissues

Gene	Squamous Cell Carcinoma (SCC)	Adenosquamous Carcinoma (ASC)	Adenocarcinoma (AC)
C7orf70	2	1	0
DNHD1	2	0	0
KPRP	1	0	1
MDM4	1	0	0
MUC6	2	1	0
OR51Q1	2	0	0
PTPRD	2	0	0
TCF4	2	0	0
TET2	4	0	0

### InDel (Insertion and Deletion) analysis

**Figure 3:**
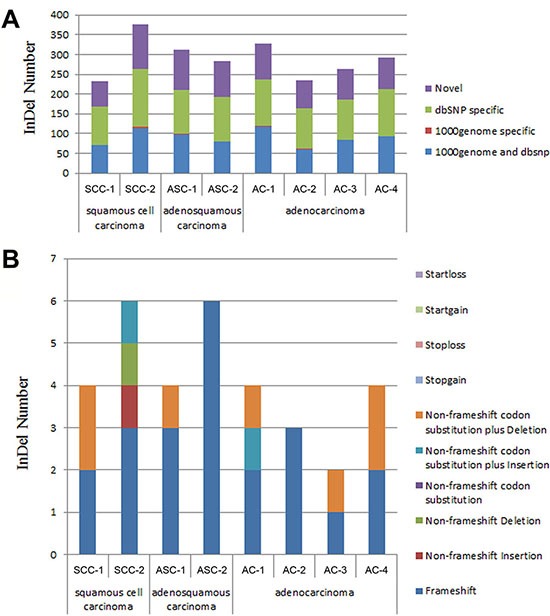
The distribution of InDels detected in pancreatic cancer tissues (**A**) Distribution of InDels for each sample in 1000 genome and dbSNP. 1000 genome and dbSNP: the number of InDels that fall into both 1000 genome and dbSNP; 1000 genome specific: the number of InDels that only fall into 1000 genome, not in dbSNP; dbSNP specific: the number of InDels that only fall into dbSNP, not in 1000 genome; The InDels that fall into neither 1000 genome nor dbSNP were considered as “novel”. (**B**) Distribution of total InDels that fall into the different categories leading to different genetic characters. Startloss: a nonframeshift insertion/deletion/substitution that leads to the immediate elimination of start codon at the variant site; Startgain: a nonframeshift insertion/deletion/substitution that leads to the immediate creation of start codon at the variant site; Stoploss: a nonframeshift insertion/deletion/substitution that leads to the immediate elimination of stop codon at the variant site; Stopgain: a nonframeshift insertion/deletion/substitution that leads to the immediate creation of stop codon at the variant site; Non-frameshift codon substitution plus Deletion: a codon substitution of one or more nucleotides plus a deletion of 3 or multiples of 3 nucleotides that do not cause frameshift changes in protein coding sequence; Non-frameshift codon substitution plus Insertion: a codon substitution of one or more nucleotides plus an insertion of 3 or multiples of 3 nucleotides that do not cause frameshift changes in protein coding sequence; Non-frameshift codon substitution: a codon substitution of one or more nucleotides that does not cause frameshift changes in protein coding sequence; Non-frameshift Deletion: a deletion of 3 or multiples of 3 nucleotides that do not cause frameshift changes in protein coding sequence; Non-frameshift Insertion: an insertion of 3 or multiples of 3 nucleotides that do not cause frameshift changes in protein coding sequence; Frameshift: an insertion/deletion/substitution of one or more nucleotides that cause frameshift changes in protein coding sequence.

A total of 232~377 InDels have been discovered for the SCC, ASC and AC of pancreatic cancers (Table 6). A percentage of 27.15% to 32.37% detected InDels were not found in the dbSNP or 1000 genome (Table 6, Figure 3A). Approximately 63~113 novel Inserts and Deletions have been found in each sample of the pancreatic carcinoma FFPE tissues (Table 6). In addition, from one to six framshift InDels were found in each FFPE sample (Table 6, Figure 3B).

**Table 6:** Summary of InDels for FFPE pancreatic SCC, ASC and AC tissues

Sample	Squamous Cell Carcinoma (SCC)		Adenosquamous Carcinoma (ASC)		Adenocarcinoma (AC)			
	SCC-1	SCC-2	ASC-1	ASC-2	AC-1	AC-2	AC-3	AC-4
Total	232	377	312	283	327	235	263	293
1000genome and dbsnp	70	113	98	80	118	61	84	93
1000genome specific	0	4	2	0	1	1	1	1
dbSNP specific	99	147	111	113	119	101	102	119
dbSNP rate	72.84%	68.97%	66.99%	68.20%	72.48%	68.94%	70.72%	72.35%
Novel	63 (27.15%)	113 (29.97%)	101 (32.37%)	90 (31.80%)	89 (27.22%)	72 (30.64%)	76 (28.90%)	80 (27.30%)
Hom	140 (60.34%)	226 (59.59%)	167 (53.53%)	154 (54.42%)	167 (51.07%)	116 (49.36%)	130 (49.43%)	151 (51.54%)
Het	92	151	145	129	160	119	133	142
Frameshift	2	3	3	6	2	3	1	2
Non-frameshift Insertion	0	1	0	0	0	0	0	0
Non-frameshift Deletion	0	1	0	0	0	0	0	0
Non-frameshift codon substitution	0	0	0	0	0	0	0	0
Non-frameshift codon substitution plus Insertion	0	1	0	0	1	0	0	0
Non-frameshift codon substitution plus Deletion	2	0	1	0	1	0	1	2
Stopgain	0	0	0	0	0	0	0	0
Stoploss	0	0	0	0	0	0	0	0
Startgain	0	0	0	0	0	0	0	0
Startloss	0	0	0	0	0	0	0	0

### Gene expression characteristics of pancreatic SCC

Besides investigation on the genomic level, we also evaluated the gene expression characteristics of pancreatic SCC. Based on data provided by Peter Bailey et al. [6], expressions of 12633 genes were found to be changed in pancreatic SCCs according to normal pancreas tissues, reaching more than 1.5-fold [6]. And 8470 of them were found to be up- or down-regulated by over 2-fold [6].

**Figure 4:**
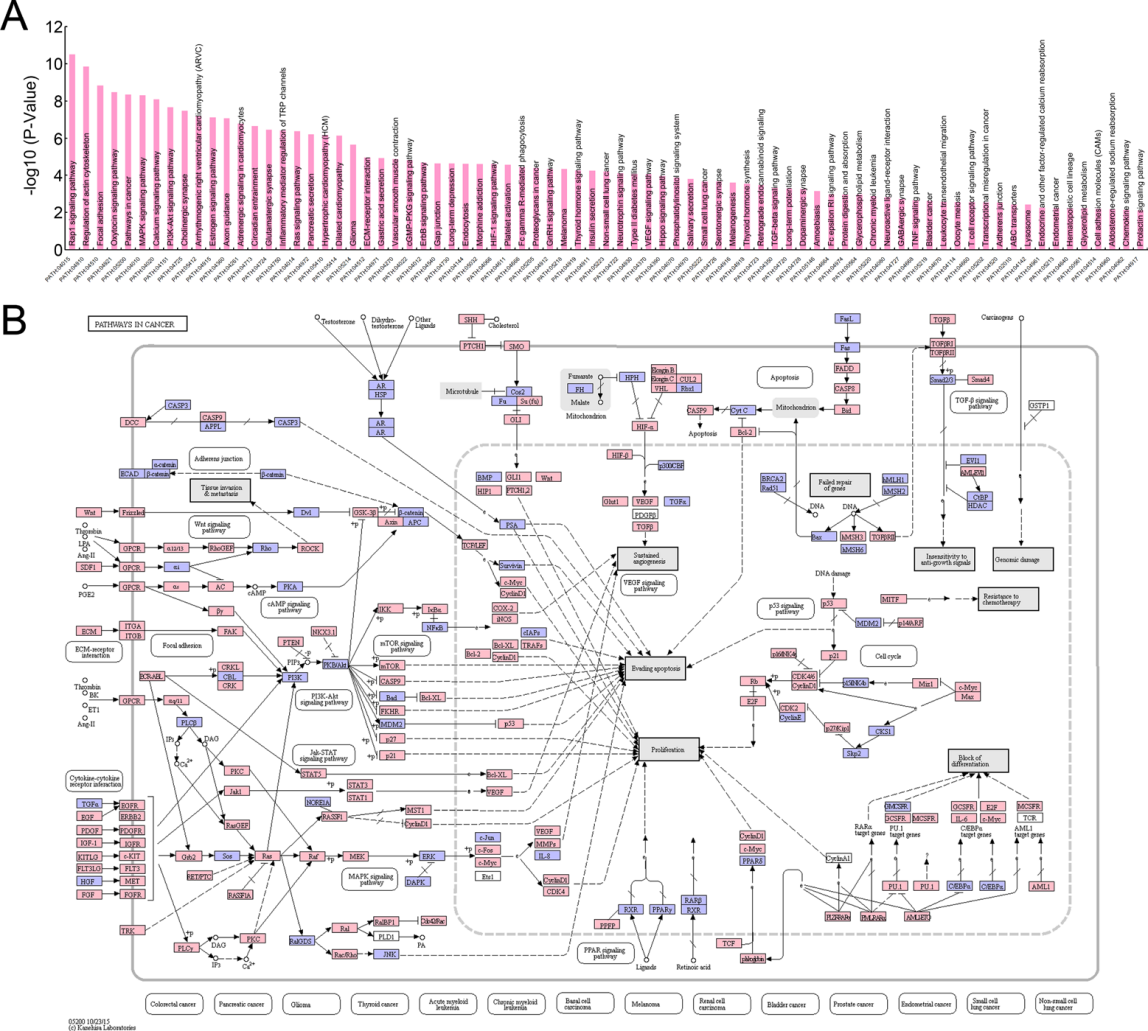
KEGG pathway assay of genes differentially expressed in pancreatic SCCs (**A**) Top pathways identifiedby using KEGG pathway assay (*P* < 0.01). (**B**) Genes involved in the cell signaling transduction of pathways in cancer (PATH:05200).

To illustrate a more detailed cell signaling transduction pathway network, these 12633 genes were subjected to KEGG pathway assay. As shown in Supplementary Table 2 and Figure 4, these genes participated in multiple pathways in cancer (PATH:05200), including PI3K-Akt signaling pathway, Focal adhesion, MAPK signaling pathway, Ras signaling pathway, VEGF signaling pathway, TGF-β signaling pathway, TNF signaling pathway, Cell adhesion molecules (CAMs), Tight junction, Wnt signaling pathway, Notch signaling pathway, Hedgehog signaling pathway, mTOR signaling pathway, et al.. Since these pathways are involved in malignant biological behaviors, such as metastasis, proliferation, evading apoptosis, angiogenesis, insensitive to anti-growth signals, resistant to chemotherapy, and stemness maintaining, et al., the aberrant expression of these genes could take part in the development and progression of pancreatic SCCs.

## DISCUSSION

Pancreas is an organ without squamous cell differentiation in its normal state. However, parenchymal squamous metaplasia is commonly detected in pancreatic specimens, and can be detected in 17% to 48% of cases [4]. Despite the relatively high frequency of squamous metaplasia, pancreatic squamous cell carcinoma (SCC) is a rare primary pancreatic malignancy, accounting for 0.05–5% of all pancreatic cancers approximately [4, 16–20]. In the present study, we screened 1033 cases of pancreatic cancer in Chinese population, and identified only 2 cases of pure SCC, accounting for only 0.19% of all cases. Therefore, pancreatic SCC is a rare subtype of pancreatic cancer worldwide. The available published data on pancreatic SCC are limited, mostly presented as individual case reports or small case series studies. As a result, the characteristics of SCC remain poorly verified. It is noteworthy that, due to its rare incidence, cases about pancreatic SCC are firstly presumed to be metastatic from other primary site [21]. Metastatic spread of primary lung or esophageal SCC sometimes has been reported to pretend to be primary pancreatic SCC [5, 22]. However, it should be noted that metastatic SCC to the pancreas is also very rare [23]. So, careful exclusion is vital before we diagnose primary SCC of pancreas.

Recently, a population-based study firstly revealed the epidemiology of primary pancreatic SCC [4]. By screening 214 patients with SCC, Makarova-Rusher OV et al. proved that the incidence rate for primary pancreatic SCC differed by age, sex, race, and ethnicity. There was a significant increase in the age-adjusted incidence rate in men relative to women (RR of 1.6). When analyzed by race, the age-adjusted incidence rates of SCC were higher in African American relative to Caucasian (RR of 1.7). At the same time, the International Agency for Research on Cancer (IARC) has reported that in the United States, the highest rates were registered among black individuals (12–15 cases per 100,000 men and 8–10 cases per 100,000 women); these rates are ~30–50% higher than in their white counterparts [1, 2]. Increased risk was also observed for older age groups. 65 years and older patients had an RR of 141.1 relative to people younger than 40 years and 5.1 when compared with age group of 40 to 64 years old. Over time, the incidence rates for SCC varied but with a significantly overall increasing trend, and the incidence rates tripled between 2000 and 2012 in the United States. The annual percent increase of SCC was of 5.5%, while the annual percent increase of AC was only 1.4% [4]. Therefore, although primary pancreatic SCC is a rare neoplasm, incidence rates for this subtype are increasing.

By now, there are only twenty reported cases of pancreatic SCC (Table 7) [5, 14–16, 18, 19, 21, 24–35], providing limited, but valuable clinical information. The clinical presentation of SCC is similar to that of AC. As the cases have been reported, thirteen patients were presented with an initial symptom of abdominal or back pain [15, 16, 18, 19, 21, 24, 27, 28, 30–32, 34, 35]. Rest of them were also referred with fatigue, anorexia, nausea, tarry stool, weight loss, or jaundice when found [5, 14, 16, 25, 26, 29, 33]. Imaging examination or laparotomy detected the mass of the pancreas and found that nine of them were in the head [5, 16, 19, 21, 25, 27, 29, 30, 32], three in the body [16, 18, 35], four in the tail [14, 28, 31, 34], and three in both body and tail [24, 26, 33]. The remaining one was not mentioned, since the patient was unresectable with liver metastasis at diagnosis [15]. Fifteen of them endured resection. Seven of the resected tumors were composed of moderately differentiated SCC [16, 19, 26, 27, 30, 33, 35]. Three were well differentiated [18, 28, 29]. One was moderately to poorly differentiated [31]. Another one was poorly differentiated SCC [21]. The rest of the cases were not mentioned. We also noticed that some specific markers might be useful for SCC diagnosis and prognosis evaluation. Most cases reported that the serum SCC-related antigen (SCC-Ag) level was often elevated, and decreased markedly after resection [33]. Previous studies also find that CD44 and its variants were good markers of squamous epithelial differentiation in several types of normal epithelium and tumors. These markers can distinguish areas of well- to moderately-differentiated squamous elements from AC elements. However, poorly differentiated tumors show an inconsistent staining pattern with CD44, so it cannot be used in poorly differentiated neoplasms [36].

**Table 7:** Reported 20 cases of pancreatic SCC in the literatures

Author	Age	Sex	Presenting symptom (s)	Location	Size (cm)	Surgery	TNM	Differentiation	Outcome	Treatment	Time to death (months)
Kodavatiganti R et. al. [4]	70	M	Weight loss, generalized itching, jaundice	Head	4.6×4.1	Pylorus-sparing pancreaticoduodenectomy	pT4N1M0, Stage III	Not known	Liver metastatic after 1 months of surgery; Death	Palliative chemotherapy after liver metastatic (cisplatin/5-FU)	8 months after surgery because of sepsis.
Ikeda A et. al. [19]	79	M	Fatigue, unintentional weight loss	Tail	5	N/A	Stage IV	Not known	Liver metastasis at diagnosis; Death	Palliative chemotherapy (S-1 100 mg/day). Four courses of S-1 tumor progressed, gemcitabine as second-line chemotherapy.	7 months after treatment
De Souza AL et. al. [20]	61	M	Anorexia, weight loss, right upper back and stomach pain	Not known	Not known	N/A	Stage IV	Not known	Liver metastasis at diagnosis; Death	Palliative chemotherapy (gemcitabine/cisplatin every 14 days for 4 months, progressed after 8 weeks, received 5-FU/leucovorin as second-line therapy for 3 months)	11 months from treatment
Ben Kridis W et. al. [21]	48	M	Epigastric pain epigastric pain	Head	4.6	N/A	Not known	Not known	Death	Chemotherapy one cycle (5-FU/cisplatin) + palliative radiotherapy	9 months
Ben Kridis W et. al. [21]	42	M	vomiting, peri umbilical pain	Body	4×3.7×3.1	Spleno-corporeo-caudal pancreatectomy	pT3N0M0, Stage IIA	Moderately differentiated (G2)	Not known	Adjuvant chemotherapy (5-FU/leucovorin)	More than 26 months
Nikfam S et. al. [23]	66	F	Epigastric pain	Body	3.5×4	N/A	Not known	Well differentiated	Death	Palliative chemotherapy (gemcitabine)	9 months after treatment
Beyer KL et. al. [24]	33	F	Upper abdominal pain	Head	10-12	Palliative bypass surgery (Roux-en-Y choledochojejunostomy and cystjejunostomy)	Not known	Moderately differentiated (G2)	9 months post-operation, she felt well and worked daily. She had no pain and was not jaundiced.	Immunotherapeutic agents	More than 9 months
Al-Shehri A et. al. [26]	48	F	Fatigue,anorexia, weight loss, nausea,vomiting, upper abdominal and back pain	Head	4.4×4.2	Palliative bypass surgery (gastrojejunostomy and choledochojejunostomy)	Stage IV	Poorly differentiated	Liver metastasis at diagnosis; Death	Palliative chemotherapy (carboplatin/gemcitabine)	3 months
Schultheis AM et. al. [29]	57	F	Postprandial epigastric pain	Body and tail	5-6	Not known	Not known	Not known	Death	Primary neoadjuvant radiochemotherapy	11 months after surgery
Kubota K et. al. [30]	73	F	Fatigue, appetite loss, jaundice	Head	8	Pancreatoduodenectomy	Not known	Not known	Not known	Not known	Not known
Aurello P et. al. [31]	58	F	Hematemesis, melena, and acute anemia, weight loss, vomiting	Body and tail	8	Total gastrectomy, left nephroadrenalectomy, distal pancreatectomy, splenectomy, andthe left colic flexure esection+Roux-en-Y esophagojejunostomy and colocolicanastomosis	pT4N0M0, Stage III	Moderately differentiated (G2)	Not known	Adjuvant chemotherapy (cisplatin/5-FU, two cycles)	Not known
Sears HF et. al. [32]	70	F	Epigastric distress, anorexia, weight loss, nauseau, vomiting	Head	6	Palliative bypass surgery (choledochojejunostomy)	Stage IV	Moderately differentiated (G2)	Porta hepatis metastasis at laparotomy; Death	Radiotherapy	6 weeks after radiotherapy
Adachi K et. al. [33]	67	F	Anorexia, back pain	Tail	6×6×7	Resected with a total gastrectomy, distal pancreatectomy and splenectomy	pT3N1M0 Stage IIB	Well differentiated (G1)	Local recurrence after 4 months of surgery; Death	Radiation therapy for local recurrence	11 months after surgery
Chen QP et. al. [34]	55	M	Jaundice, pale-colored stool, anorexia, weight loss	Head	4×5×5	Pancreaticoduodenectomy,resection of the lateral wall of the portal vein and the left lobe of liver	Not known	Well-differentiated	Death	Not known	10 months after surgery
Terada T et. al. [35]	69	F	Abdominal pain, jaundice	Head	5×5×6	Pancreaticoduodenal resection and cholecystectomy	Not known	Moderately differentiated (G2)	Death	Not known	3 months after surgery
Brown HA et. al. [36]	56	M	Back pain	Tail	6	Resected with bloc distal pancreatectomy,splenectomy, partial gastrectomy, and left adrenalectomy	pT2N0MX Stage IB	Moderately to poorly differentiated (G2-3)	Liver metastatic after 3 months of surgery; Death	Chemotherapy after liver metastatic	Not known
Rana SS et. al. [37]	50	M	Upper abdominal pain radiated to the back, vomiting, jaundice	Head	Not known	Palliative bypass surgery (jejunostomy)	Not known	Not known	Not known	Palliative treatment	Not known
Minami T et. al. [38]	62	M	Fatigue, fever, tarry stool	Body and tail	8	Distal pancreatectomy, total gastrectomy, splenectomy, and partial colectomy	pT3N0M0 Stage IIA	Moderately differentiated (G2)	Not known	Not known	More than 16 months
Lai LH et. al. [39]	76	F	Dull epigastric pain with radiation to the back, weight loss	Tail	5	N/A	Stage IV	Not known	Liver metastasis at diagnosis	Not known	Not known
Bralet MP et. al. [40]	68	F	Abdominalpain, weight loss	Body	4	Distal pancreatectomy, cholecystectomy	Not known	Moderately differentiated (G2)	Not known	Not known	More than 8 months

Surgery is still the only relatively efficient cure for this rare but virulent disease currently. However, most patients could only receive palliative bypass surgery when they were diagnosed. Makarova-Rusher OV et al. reported that only a small percentage of patients with SCC (10.3%) could receive potentially curative surgery, which improved survival [4]. The median survival, 1-year survival, and 2-year survival of recectable SCC were 10, 45.3 and 35.2 months, while that of unrecectable cohort were only 3, 10.3 and 3.7 months. For patients who received complete resections, there were no significant differences in OS of SCC and AC [4]. Other treatments for this disease include systemic chemotherapy, radiotherapy and/or immunotherapy. However, for those unresectable subjects, survival of SCC was worse than that of AC. The median survival, 1-year survival, and 2-year survival of unresectable AC were 5, 17.9 and 5.1 months, which were better than that of unresectable SCC [4]. Cisplatin was used in combinations with 5-FU (three cases) [5, 16, 26], or gemcitabine (one case) which progressed after 8 weeks and then received 5-FU/leucovorin as second-line therapy for 3months [15]. Gemcitabine has been used solo (two cases) [14, 18] or in combination with carboplatin (one case) [21]. Immunotherapy was used in one patient who had a good prognosis after palliative bypass surgery [19]. S-1 (100 mg/day) was also proved to be effective in one patient in the first four courses [14]. One patient who underwent spleno-corporeo-caudal pancreatectomy with pancreatic stump sutures and then received chemotherapy according to the FUFOL regiment (5-FU/leucovorin) was in complete remission after a follow-up period of 26 months [16]. Radiotherapy combined with chemotherapy was also used in some cases. However, there is still no standard therapy regimen established so far.

Cancers containing both squamous and glandular elements are found in many organs, like lung, esophagus, etc. Taking AC and SCC of lung as an example, previous studies proved that AC and SCC present significant differences at both expression and genomic levels [37–42]. Despite the low incidence of pancreatic SCC, accumulation of tissue samples in the past decades has led to systematic investigations in this rare subtype. After screening 25 SCC samples by using RNA-seq and 71 SCC samples by using mRNA microarray, Peter Bailey et al. identified four core gene expression programmes characterized pancreatic SCC, including gene networks involved in inflammation, hypoxia response, metabolic reprogramming, TGF-β signalling, MYC pathway activation, autophagy and upregulated expression of TP63ΔN and its target genes [6]. By using KEGG pathway assay, we illustrated the participation of these differentially expressed genes in cell signaling transduction network in cancer (Figure 4).

To explore the genomic characteristics of SCC, we used a 137-cancer-related gene panel for in-solution hybrid capture accompanied by Illumina paried-end sequencing. For some well-known pancreatic cancer biomarkers [43], we have detected the common and different SNVs in pancreatic ACs, ASCs and SCCs, respectively. For instance, the oncogene KRAS encodes for the membrane-bound GTP-binding protein, which is involved in the signaling pathways mediated by growth factor. In 80%~90% of patients with pancreatic cancer, KRAS gene is frequently detected to be mutated at codon 12 and sometimes mutated at codons 13 or 61 [44–47]. In the present study, for the oncogene KRAS, we have detected Gly12Arg mutation in pancreatic ACs, Gly12Asp mutation in SCC of pancreas, while no mutation of KRAS has been detected in pancreatic ASCs (Table 3). TP53 is encoded by p53 gene which is a tumor suppressor gene mutated in the human cancers [48]. In our analysis, TP53 possessed the same mutation (Pro72Arg) in the three groups. SMAD4 is a tumor suppressor encoded by the *SMAD4* gene located on chromosome 18q. SMAD4 has been detected to possess mutations of Pro292Ser and Arg496His in ASCs and SCCs, respectively, and no mutation of SMAD4 was detected in pancreatic ACs (Table 3). BRCA2 is involved in the transcriptional regulation, cell growth, chromatin remodeling and DNA damage repair [49–54]. Pancreatic ACs, ASCs and SCCs shared the common mutation Val2466Ala, while each of them also have some specific mutations (Table 3). The epidermal growth factor receptor (EGFR) is a glycoprotein involved in several human cancers [55]. We detected a common mutation: Arg521Lys of EGFR in all the three types of tissues, while only ACs and ASCs possessed the Leu861Glu mutation (Table 3).

Besides the common and specific mutations for the studied biomarkers of pancreatic cancer, we also found some other new specific mutated genes in either pancreatic ACs or SCCs (Table 4, Table 5). Nine mutated genes (ABCB1, CSF1R, CYP2C18, FBXW7, ITPA, KIAA0748, SOD2, SULT1A2, ZNF142) did not exist in SCC samples (Table 4), among which four genes (ABCB1, CYP2C18, SOD2, ZNF142) were mutated exclusively in AC tissues (Highlighted in red in Table 4). Some of these genes are known to be involved in the regulation of oxidative stress cell death or intrinsic apoptotic signaling pathway (FBXW7, SOD2) and positive regulation of protein tyrosine kinase activity (CSD1R, FBXW7). ABCB1 is involved in multidrug resistance. The protein encoded by this gene is an ATP-dependent drug efflux pump for xenobiotic compounds with broad substrate specificity. It is responsible for decreased drug accumulation in multidrug-resistant cells and often mediates the development of resistance to anticancer drugs. CYP2C18 belongs to cytochrome P450 2C subfamily, which is involved in cancer susceptibility risk [56]. ITPA controls the level of nucleotides including ITP and dITP, which exist in all cells. It has been suggested that the function of this ubiquitous protein family is the elimination of minor potentially mutagenic or clastogenic purine nucleoside triphosphatases from the cell. The mRNA expression level of ITPA was proved to be higher in tumor cells than those in normal tissues, showing preferentially expressed in tumor cells [57]. KIAA0748, also known as TESPA1, is expressed in lymphocytes and is phosphorylated in response to store-operated calcium entry. SULT1A2 which drastically reduces its affinity for the substrate, is supposed to be associated with bladder cancer and breast cancer [58, 59]. ZNF142 is related to transcriptional regulation, controlling cell growth, proliferation, differentiation, and apoptosis. We also detected nine mutated genes (C7orf70, DNHD1, KPRP, MDM4, MUC6, OR51Q1, PTPRD, TCF4, TET2) in SCCs or ASCs (Table 5), among which four genes (DNHD1, OR51Q1, PTPRD, TCF4) were mutated only in SCCs (Highlighted in red in Table 5). DNHD1 and OR51Q1 are known very little, but a clinical genomics study shows that DNHD1 is harbored independent homozygous mutations in patients with overlapping phenotypes [60]. PTPRD is known to regulate a variety of cellular processes including cell growth, differentiation, mitotic cycle, and oncogenic transformation. Studies also identified deletion of PTPRD in head and neck SCC [61]. TCF4 encodes transcription factor 4, and is closely related to the canonical Wnt pathway, which plays key roles in development, tissue homeostasis, and cancer. Intriguingly, TET2 is known to be involved in histone H3K4 methylation and DNA demethylation, suggesting that this gene might work in SCC through epigenetic mechanisms. Taking together with previous study [6], the development and progression of pancreatic SCC could be driven by both specific gene mutations and uncontrolled gene expression network.

Previous studies provided many theories concerning the histogenesis of squamous elements of pancreatic SCC. These theories may be summarized as follows: (1) heterotopic or metaplastic squamous epithelium undergoes malignant change due to chronic inflammation, (2) preexisting adenocarcinoma undergoes malignant transformation (malignant metaplasia) into squamous cell carcinoma, (3) SCC that originates from a mixed ASC in which the glandular components are not visible, and (4) a primitive cell capable of differentiating into either squamous or glandular cell carcinoma undergoes malignant change [22, 28, 62–65]. Based on the gene expression patterns, Peter Bailey et al., recently found that inflammatory pathway played a vital role in the gene networks of pancreatic SCC [6]. Therefore, this investigation provided the first direct evidence that chronic inflammation could be involved in the development and progression of pancreatic SCC. Since inflammation has been proved to be able to promote the growth and metastasis of pancreatic cancer [66], the participation of inflammatory pathway in pancreatic SCC may explain why this subtype has the worst outcome, comparing to other subtypes [6].

Limited by the rareness of pancreatic SCC tissue samples, our study is just the tip of the iceberg, and further researched need to be undertaken in the future. To the best of our knowledge, our study was the first investigation providing the list of differential mutations in pancreatic AC and SCC, which could provide valuable information for understanding the pathogenesis of pancreatic SCC and for further targeted drug development.

## MATERIALS AND METHODS

### Tissue specimens

The pancreatic tissues were obtained from patients with adenocarcinoma (AC), squamous cell carcinoma (SCC) and adenosquamous carcinoma (ASC) who underwent the surgery of pancreaticoduodenectomy. The diagnosis of AC, SCC and ASC of the pancreas was confirmed by pathological examination. The Formalin Fixed Paraffin Embedded (FFPE) tissue specimens of SCC (2 patients) and ASC (2 patients) were provided by the First Affiliated Hospital of Soochow University and Renji Hospital of Shanghai, while those of AC were taken from 4 patients admitted to the Second Affiliated Hospital of Soochow University. The tissues were fixed in 10% neutral buffered formalin and embedded in paraffin with the FFPE protocol. All human tissue samples were obtained and handled in accordance with an approved Institutional Review Board application (the Committee on Medical Ethics, the First Affiliated Hospital of Soochow University). All experiments were performed in accordance with relevant guidelines and regulations of the Committee on Medical Ethics of the First Affiliated Hospital of Soochow University. All the patients signed an informed consent for participation of the study and the use of their biological tissues. Primary SCC of pancreas was pathologically diagnosed on the basis of finding definite intercellular bridges and/or focal keratin peal formation in the tumor cells.

### Immunohistochemistry

All resection specimens were fixed in 10% buffered formalin and paraffin-embedded by routine processing. Sections were cut at 4-μm thickness, heated at 60°C for 30 min, then deparaffinized and hydrated through a series of xylene and alcohol baths. The slides were microwaved with antigen retrieval solution (citrate buffer, pH 6.0, containing 0.3% trisodium citrate and 0.04% citric acid) for 5 min. After replenishment of this solution, the slides were microwaved again for 5 min and then allowed to cool for 20 min. The sections were then rinsed in PBS and immersed in 3% H_2_O_2_ for 15 min to block the endogenous peroxidase. Thereafter, the sections were incubated with 10% bovine serum albumin at room temperature for 1 h to block nonspecific antibodies. Immunohistochemical staining was performed with mouse anti-P63 antibody (Dako, Carpenteria, CA, USA) or mouse anti-CK5/6 antibody (Dako) at room temperature for 2 h. After incubation with the corresponding secondary antibodies for 20 min, the bound complex was visualized using a SuperPicture Polymer Detection Kit (Life Technologies, Valencia, CA, USA).

### Extraction of DNA

Genomic DNA was extracted from the FFPE samples using the QIAamp DNA FFPE Tissue Kit (Cat No. 56404, Qiagen, Valencia, CA, USA) according to the manufacturer's protocols. 1 μl of DNA was used to measure DNA concentration on a Qubit 2.0 Fluorometer (Life Technologies), and the quality control of DNA was done by running the DNA on a 1% agarose gel.

### Library construction

220–320ng of DNA for each sample was used to construct the pre-captured DNA library using DNA Seq Library Preparation Kit-Illumina Compatible (K02422, Gnomegen, San Diego, CA, USA) according to the manufacturer's protocol. The fragmented DNA was subsequently end repaired, ligated to adaptors and subjected to PCR amplification with 9 or 11 amplification cycles according to the manufacturer's instructions with several purification steps to get library products with different indexes (K02422, Gnomegen). The pre-captured library was purified with Gnome Size Selector (R02424, Gnomegen) and quantified with a Qubit 2.0 Fluorometer (Life Technologies), and quality control was performed by running the purified library on a 1% agaroase gel.

### In-solution hybrid capture and illumina sequencing

The pre-captured DNA was hybridized to the 137 cancer gene probes (Roche NimbleGen, Madison, Wisconsin, USA) at 47°C for 64–72 h, which can enrich the exonic sequences of 137 cancer-related genes according to the manufacturer's protocol from NimbleGen SeqCap EZ Library SR User's Guide v3.1 (Roche NimbleGen). The 137 genes that we chose are known to have somatic genomic variants in cancer [11–13]. These genes contain 2372 exons that encode 433,159 bases. The exome-enriched libraries were sequenced on the Illumina HiSeq 2000 platform with 1000× sequencing depth, and paired-end reads with an average size of 125 bp (PE125) were generated.

### Bioinformatic analysis

The sequencing reads were mapped to the reference human genome (UCSC hg19) with BWA [67, 68]. We used the quality threshold (-q35) and mismatch penalty (-M) as 3 and -d as 10 to map the unique reads. SNVs (single-nucleotide variants) and InDels (Insertions and Deletions) were subsequently called using the Genome Analysis Toolkit [69] with default parameters [70]. The functions of the specific mutated genes in either AC or SCC were annotated and analyzed with the EnrichR tool [71]. Briefly, the list of the specific mutated genes was submitted to EnrichR website: http://amp.pharm.mssm.edu/Enrichr/, and searched for the databases of Gene Ontology (GO) involving molecular functions or biological processes. Kyoto Encyclopedia of Genes and Genomes (KEGG) pathway database (http://www.genome.jp/kegg/pathway.html) was used for pathway analysis and mapping [72].

## SUPPLEMENTARY MATERIALS TABLES




