# Supplementary material for: Genomic characteristics of pancreatic squamous cell carcinoma, an investigation by using high throughput sequencing after in-solution hybrid capture
Source: Oncotarget. 2017 Jan 16;8(9):14620–35. doi: 10.18632/oncotarget.14678 (PMC5362430; doi:10.18632/oncotarget.14678)
Supplement: Supplementary file 1 [file oncotarget-08-14620-s001.pdf]

Genomic characteristics of pancreatic squamous cell carcinoma, an investigation by using high throughput sequencing after in-solution hybrid capture

Supplementary Materials

Supplementary Table 1: List of 137 cancer-related gene panel

ABL1	ABL2	AKT1	AKT2	AKT3	ALK	APC	ATM
AURKA	BCL2	BRAF	BRCA1	BRCA2	CCND1	CCNE1	CDC73
CDH1	CDK4	CDK6	CDK8	CDKN1A	CDKN2A	CEBPA	CHEK1
CHEK2	CREBBP	CRKL	CSF1R	CTNNB1	EGFR	EPHA3	EPHA5
EPHB6	ERBB2	ERBB3	ERBB4	FBXW7	FGFR1	FGFR2	FGFR3
FGFR4	FHIT	FKBP9	FLT1	FLT3	FLT4	MTOR	GNAQ
GNAS	GUCY1A2	HNF1A	HRAS	HSP90AA1	IDH1	IDH2	IGF1R
IKBKE	IKZF1	JAK2	JAK3	KDR	KEAP1	MTUS2	RPTOR
KIT	KRAS	MAP2K1	MAP2K2	MAP2K4	MCL1	MDM2	MDM4
MEN1	MET	MITF	MLH1	MLL	MPL	MRE11A	MSH2
MSH6	MYC	MYCL1	MYCN	NF1	NF2	NKX2-1	NOTCH1
NOTCH2	NOTCH3	NOTCH4	NPM1	NRAS	NTRK1	NTRK2	NTRK3
PAX5	PDGFRA	PDGFRB	PDPK1	PIK3CA	PIK3R1	PTCH1	PTEN
PTK2B	PTPN11	PTPRD	RAF1	RB1	REL	RET	RICTOR
RUNX1	RUNX1T1	SMAD2	SMAD3	SMAD4	SMARCA4	SMARCB1	SMO
SOCS1	SRC	STK11	SUFU	TCF4	TERT	TET2	TGFBR2
TNFAIP3	TOP1	TP53	TSC1	TSC2	TSHR	VHL	WT1
ZNF668							

Supplementary Table 2: Genomic characteristics of pancreatic squamous cell carcinoma, an investigation by using high throughput sequencing after in-solution hybrid capture. See Supplementary_Table_2
